# A Protective Role for Androgen Receptor in Clear Cell Renal Cell Carcinoma Based on Mining TCGA Data

**DOI:** 10.1371/journal.pone.0146505

**Published:** 2016-01-27

**Authors:** Hongjuan Zhao, John T. Leppert, Donna M. Peehl

**Affiliations:** 1 Department of Urology, Stanford University School of Medicine, Stanford, California, United States of America; 2 Veterans Affairs Palo Alto Health Care System, Palo Alto, California, United States of America; University of Central Florida, UNITED STATES

## Abstract

Androgen receptor (AR) is expressed in normal murine and human kidneys of both genders, but its physiologic role is uncertain. Several studies showed loss of AR in renal cell carcinoma (RCC) in conjunction with increasing clinical stage and pathological grade, but others found that higher AR expression correlated with worse outcomes. Limited functional studies with renal cell lines suggested tumor-promoting activity of AR. In this study, we queried transcriptomic, proteomic, epigenetic and survival data from The Cancer Genome Atlas (TCGA) to evaluate AR expression and its association with overall survival in three subtypes of RCC (clear cell [ccRCC], papillary [pRCC], and chromophobe [chRCC]). We found that although there was no significant difference in AR mRNA expression in ccRCC of males vs. females, AR protein expression in ccRCC was significantly higher in male compared to female patients. More importantly, higher expression of AR at both transcript and protein levels was associated with improved overall survival in both genders with ccRCC, but did not predict survival of either gender with pRCC or chRCC. Genes whose transcript levels were associated with AR mRNA levels significantly overlapped between ccRCC and pRCC, but not with chRCC, suggesting a similar transcriptional program mediated by AR in ccRCC and pRCC. Ingenuity pathway analysis also identified overlapping pathways and upstream regulators enriched in AR-associated genes in ccRCC and pRCC. Hypermethylation of CpG sites located in the promoter and first exon of AR was associated with loss of AR expression and poor overall survival. Our findings support a tumor suppressor role for AR in both genders that might be exploited to decrease the incidence or progression of ccRCC.

## Introduction

In the U.S., renal cell carcinoma (RCC) is the 6^th^ leading cause of cancer deaths in men and the 8^th^ leading cause in women, and the incidence of RCC continues to rise [1]. Surgery (radical or partial nephrectomy) is the standard treatment for primary RCC, while seven targeted therapies are FDA-approved for metastatic RCC [2]. Although these therapies have extended survival of patients with advanced RCC, the response rate is low and relatively short-lived and the 5-year survival rate of patients with metastatic RCC remains at less than 10%. More efficacious approaches are needed for treatment of metastatic disease as well as for neoadjuvant and adjuvant therapy of localized RCC [3].

The incidence of RCC is ~60% higher in men than in women, suggesting a possible sex-linked risk factor [4]. The androgen receptor (AR) signaling pathway is one candidate. AR is expressed in the distal and proximal tubules of male and female human kidneys and may play a role in renal sodium and calcium excretion and maintaining blood pressure [5]. Previous studies also suggested that the murine kidney is highly androgen-responsive [6,7], and AR signaling regulates metabolic processes, especially lipid and nitrogen metabolism, in the normal murine kidney [8].

Loss or gain of AR function has been implicated in cancers arising from AR-expressing tissues, including RCC. However, the role of AR in the development and progression of RCC is not clear. Several studies suggested that AR protein expression was significantly associated with low stage, well differentiated tumors and favorable prognosis [9–11]. For example, AR expression in metastatic RCC was less frequent than in primary RCC, with Zhu et al. finding no AR expression in 16/16 metastases [10], Brown et al. finding 1/5 AR-positive [12], and Williams et al. finding 35/126 AR-positive [11]. The association of loss of AR with increasing cancer aggressiveness in these studies suggests a tumor suppressor role for AR in RCC, yet two recent studies found that increased expression of AR mRNA associated with poor prognosis [13,14]. Furthermore, certain functional studies with renal cell lines suggested a tumor promoting role for AR [13,15].

Resolution of the functional role of AR in RCC has clinical implications. If AR suppresses malignancy, then strategies to maintain or restore AR in RCC could have therapeutic efficacy. Alternatively, if AR promotes RCC growth, then second-generation anti-androgen therapies that have been developed for prostate cancer might prove useful in treating RCC [16]. Here, we analyzed data extracted from The Cancer Gene Atlas (TCGA) to gain additional insight into the role of AR signaling in RCC.

## Materials and Methods

### Ethics statement

We analyzed raw data that is published in The Cancer Genome Atlas (TCGA).

### TCGA

Gene expression data by RNA-seq for cohorts of 473 ccRCC, 120 pRCC and 48 chRCC cases that have clinical outcome data available were extracted from TCGA. These cohorts included ~ 20,500 data points each for ccRCC, pRCC and chRCC. Clinical information for each patient including survival status, time to last follow-up, and gender was also extracted from TCGA. AR protein expression data and matching clinical information were retrieved from TCGA for 428 ccRCC patients. AR DNA methylation data and matching clinical information were retrieved from TCGA for 208 ccRCC patients. Mean methylation levels of 11 CpG sites located in AR promoter and first exon were used in survival analysis.

### Survival analysis

Patients were separated into two groups based on the mean levels of AR mRNA expression, protein expression, or DNA methylation. Patients were also grouped into two clusters using hierarchical clustering analysis of AR-associated gene expression (see below). Kaplan-Meier analysis was performed using XLSTAT in Excel.

### AR-associated gene expression

Each extracted gene expression matrix was analyzed to identify genes that were highly co-expressed with AR across the dataset. Pearson correlation coefficients (CC) between AR expression and that of every gene in the dataset were calculated. AR co-expressed genes were identified using the cutoff of absolute CC value of >0.5.

### Hierarchical clustering analysis

One hundred ninety-one genes whose mRNA expression was positively associated with AR mRNA expression and 8 genes whose mRNA expression was negatively associated with AR mRNA expression, identified as described above, were used in hierarchical clustering analysis. Data were log transformed and mean centered for each gene. Average linkage clustering was performed using Cluster Software (http://www.eisenlab.org/eisen/?page_id=42). Results were visualized using TreeView software (http://www.eisenlab.org/eisen/?page_id=42).

## Results

### Prognostic value of AR expression in RCC

**Fig 1:**
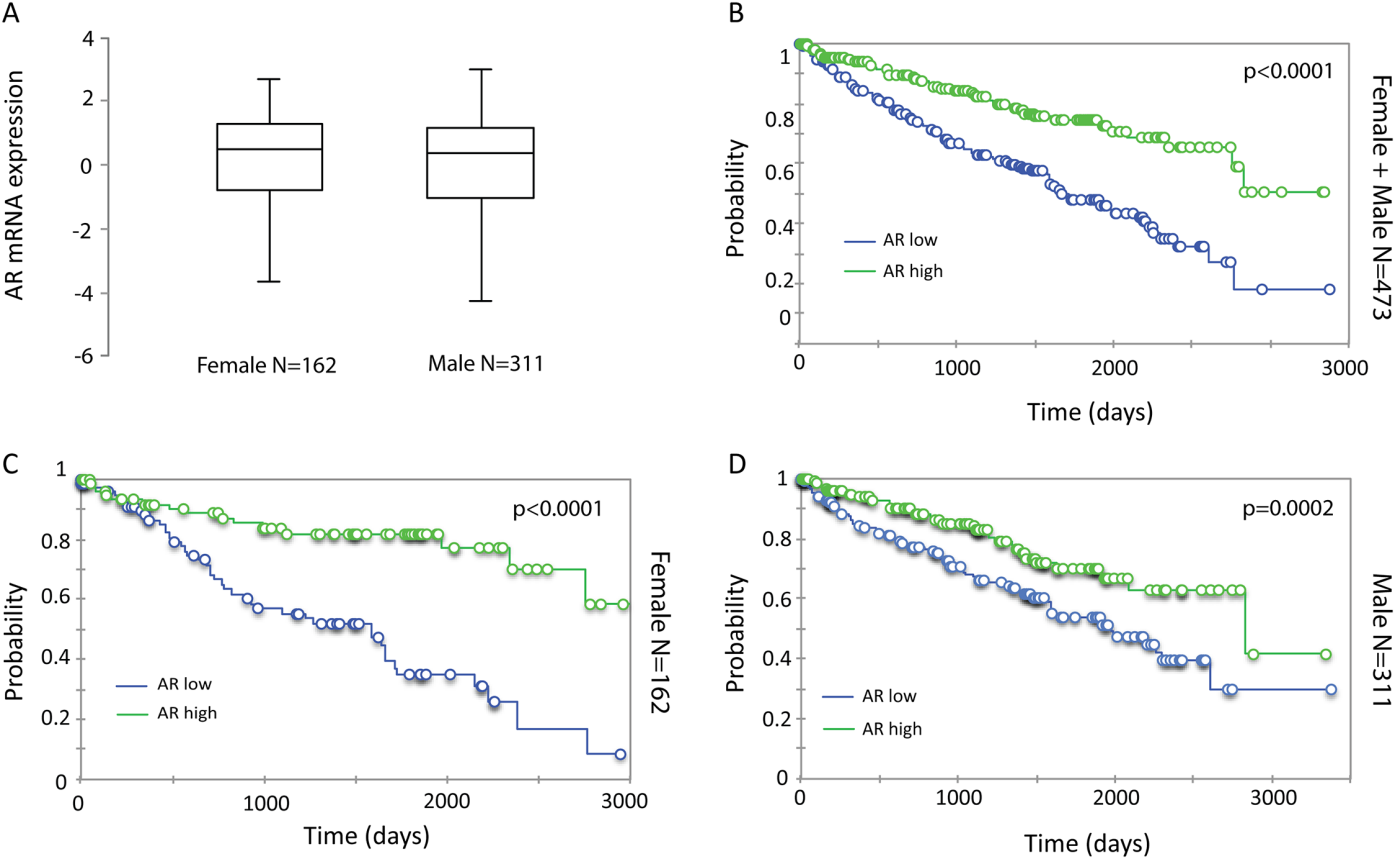
mRNA expression of AR and its association with survival in ccRCC. (A) Box plot of log2 transformed and mean centered AR mRNA levels in females and males. No significant difference was observed by student’s t-test. (B-D) Kaplan-Meier analysis of AR mRNA expression and overall survival in all cases (B), females (C) and males (D).

AR mRNA expression levels and clinical follow-up of 473 cases of ccRCC, the most common subtype of RCC, were obtained from TCGA. No significant differences in AR expression were observed between men and women (Fig 1A). Cases were assigned into AR-high or AR-low groups using median AR mRNA expression as cutoff. Time to death was plotted in a Kaplan-Meier curve for those cases exhibiting expression of AR transcripts above the median (n = 236) and equal to or below the median (n = 237) level of expression. The curves were significantly different (p<0.0001), with those cases have higher expression of AR surviving longer (Fig 1B). AR was similarly protective against poor outcome when calculated for females (n = 162) (Fig 1C) and for males alone (n = 311) (Fig 1D), with a higher impact in females (p<0.0001) than in males (p = 0.0002), demonstrating that AR mRNA expression was associated with favorable outcome in this cohort of ccRCC patients.

**Fig 2:**
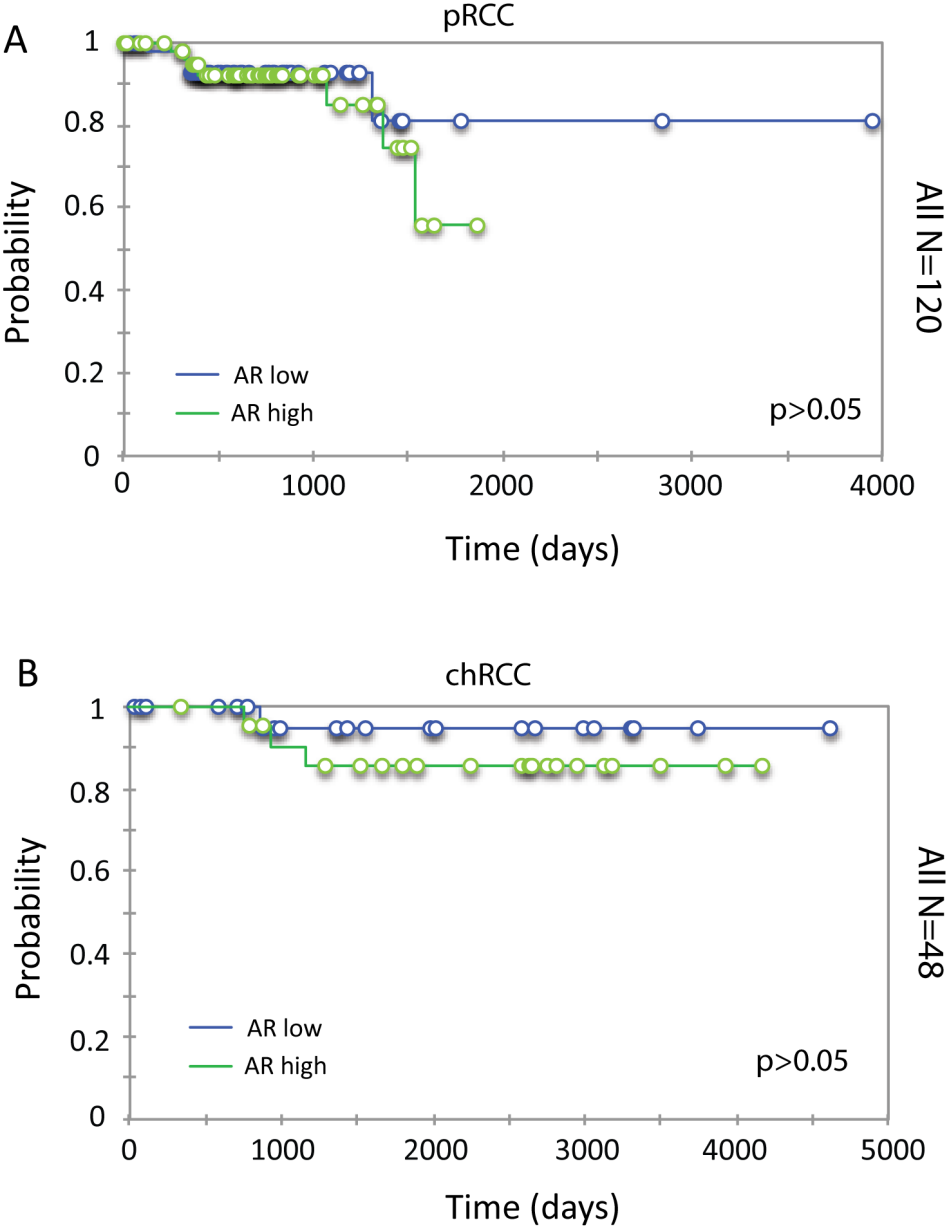
Association of AR mRNA expression with survival in pRCC and chRCC. Kaplan-Meier analysis of AR mRNA expression and overall survival in pRCC (A) and chRCC (B) patients.

Similar analyses were carried out for pRCC (n = 120) and chRCC (n = 48). In neither subtype of RCC was AR expression associated with time to death (Fig 2), and this was also the case when males and females were considered separately (not shown), possibly due to the fact that the number of patients with poor outcomes in these two subtypes was relatively small, especially for chRCC. Indeed, power analysis revealed a 65% and 17.4% power of detecting a difference in survival with an alpha error of 0.05 for pRCC and chRCC respectively. Alternatively, the differences in the relationships of AR expression and survival in the subtypes of RCCs may be attributable to distinctive functions of AR in these subtypes.

**Fig 3:**
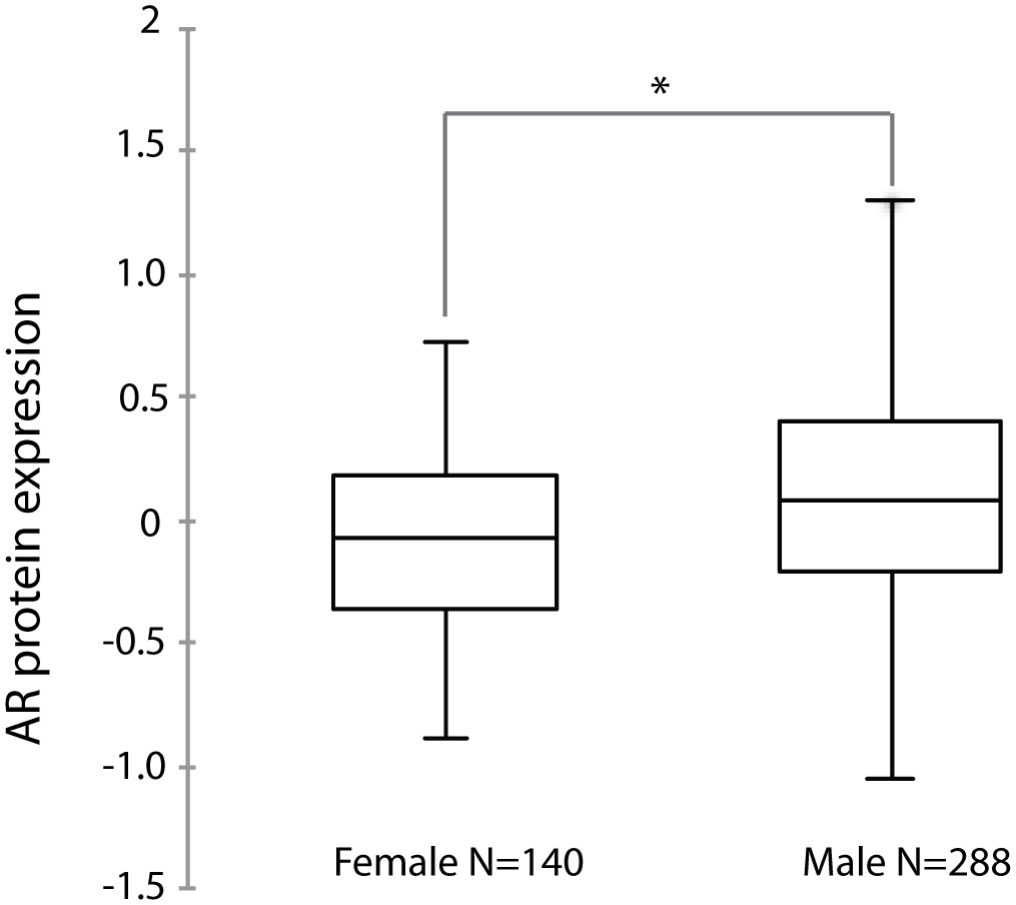
Box plot of AR protein expression in male and female ccRCC patients. * indicates statistical significance (p<0.05) by student’s t-test.

**Fig 4:**
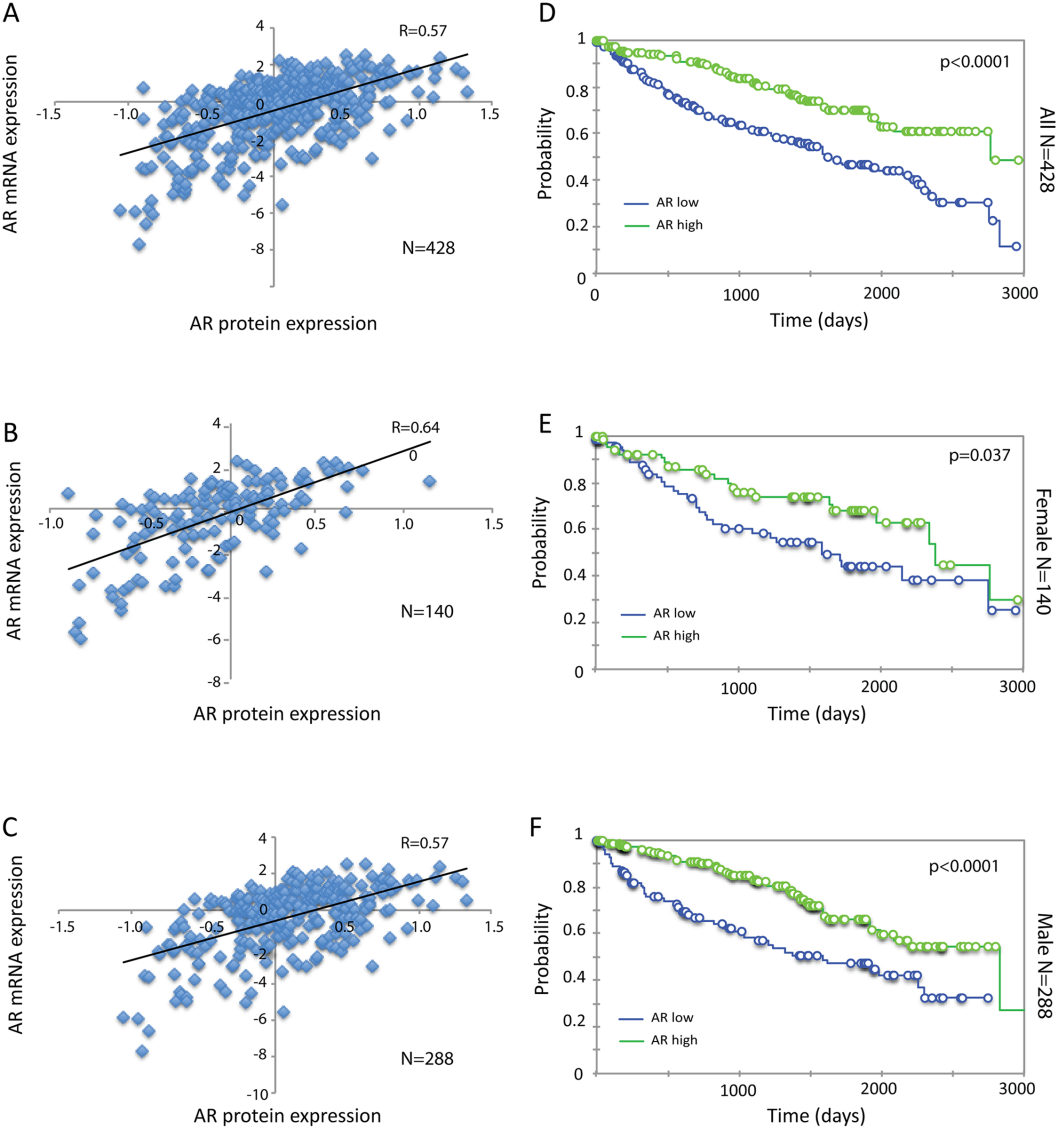
Protein expression of AR and its association with survival in ccRCC. (A-C) Correlation of AR protein and log2 transformed and mean centered mRNA expression level shown by scatter plot in all cases (A), females (B) and males (C). (D-F) Kaplan-Meier analysis of AR protein expression and overall survival in all cases (D), females (E) and males (F).

AR protein expression determined by Reverse Phase Protein Array (RPPA) for 428 cases of ccRCC with matched AR mRNA expression was retrieved from TCGA. Consistent with the probability that males have higher testosterone levels than females and that binding of androgen stabilize AR protein [17], AR protein showed significantly higher expression in cancers of male compared to female patients (Fig 3). AR mRNA expression showed a positive correlation with protein expression (R = 0.57) in all 428 cases (Fig 4A) and when female and male patients were analyzed separately (R = 0.64 and 0.57, respectively, Fig 4B and 4C). There are some cases that showed negative correlations between AR protein and mRNA levels, which may indicate the post-transcriptional processing of RNA. AR protein expression was associated with outcome when calculated for all 428 cases (Fig 4D), and for females (n = 140) (Fig 4E) and males (n = 288) (Fig 4F) calculated separately. Interestingly, the predictive power of AR protein expression was higher in males (p<0.0001) than in females (p = 0.037). These results suggest that higher AR protein expression predicts longer survival in ccRCC. Indeed, AR is identified as the 10^th^ most significant gene whose protein expression is positively correlated with survival (Table 1) using Wald's test in univariate Cox regression analysis available at Broad Institute TCGA GDAC (http://firebrowse.org/?cohort=KIRC#).

**Table 1:** List of top 10 genes whose protein expression levels are significantly associated with Time to Death by Cox regression test^a^.

Gene Symbol	Hazard Ratio	Wald_P	Q^b^	C_index
*SRC*	0.54	7.76E-12	1.20E-09	0.369
*VASP*	4.1	1.75E-11	1.40E-09	0.634
*CDKN1A*	6.1	6.41E-11	3.30E-09	0.624
*GAB2*	0.5	1.69E-10	6.50E-09	0.353
*ACACA*	2.6	5.85E-10	1.80E-08	0.63
*MAPK1*	0.6	1.26E-09	2.90E-08	0.352
*PRKAA1*	0.46	1.30E-09	2.90E-08	0.372
*CTNNA1*	0.2	1.50E-09	2.90E-08	0.361
*CCNB1*	1.75	2.04E-09	3.50E-08	0.581
*AR*	0.33	2.72E-09	4.20E-08	0.352

^a^from http://firebrowse.org/?cohort=KIRC#.

^b^For multiple hypothesis correction, Q value is the False Discovery Rate (FDR) analogue of the P value, defined as the minimum FDR at which the test may be called significant.

### Genes whose expression correlated with AR expression in RCC

To identify putative targets of AR signaling in RCC, the correlation of AR expression with every gene in the TCGA dataset was measured by calculating Pearson’s correlation coefficient. Genes with either a positive correlation coefficient (CC) >0.5 or a negative CC <-0.5 are listed in S1 Table. For ccRCC, 191 genes were positively correlated and 8 were negatively correlated with AR expression. Two hundred and fifty-two genes positively correlated with AR expression in pRCC, and zero genes negatively correlated. Genes positively and negatively correlated with AR expression in chRCC numbered 170 and 0, respectively. There was considerable overlap in AR-correlated gene expression between ccRCC and pRCC (120 out of 191 and 252, 63% and 48%, respectively), but not between chRCC and ccRCC or pRCC (Table 2), perhaps indicating a different role for AR in chRCC compared to the other subtypes.

**Table 2:** Number of AR-associated genes in different subtypes of RCC.

	ccRCC	pRCC	chRCC
**Positively associated**	191	252	170
**Negatively associated**	8	0	0
**Positively-associated overlapping genes**			
**ccRCC**		120	10
**pRCC**			17

When genes associated with AR expression were queried against ARGD [Androgen responsive gene database (http://argdb.fudan.edu.cn/geneshow_basic.php)], which contains 3321 androgen responsive protein coding genes (experimentally proved to be regulated by androgen at the RNA or protein level or proved to contain one or more transcription regulatory regions that could be bound by AR or mediate the response of reporter genes to androgen), 81 out of 191 (50%) or 2 out of 8 (25%) genes positively or negatively associated with AR in ccRCC, respectively, were found in the database. Similarly, 79 out of 252 (31%) genes positively associated with AR in pRCC were found in the database. Fifty-six out of 120 (47%) genes positively associated with AR overlapping between ccRCC and pRCC were found in the database. Finally, 38 out of 170 (22%) genes positively associated with AR in chRCC were found in the database. These results suggest that AR plays an active role in regulating gene expression in all three subtypes of RCC and that it mediates a similar transcriptional program in ccRCC and pRCC.

**Fig 5:**
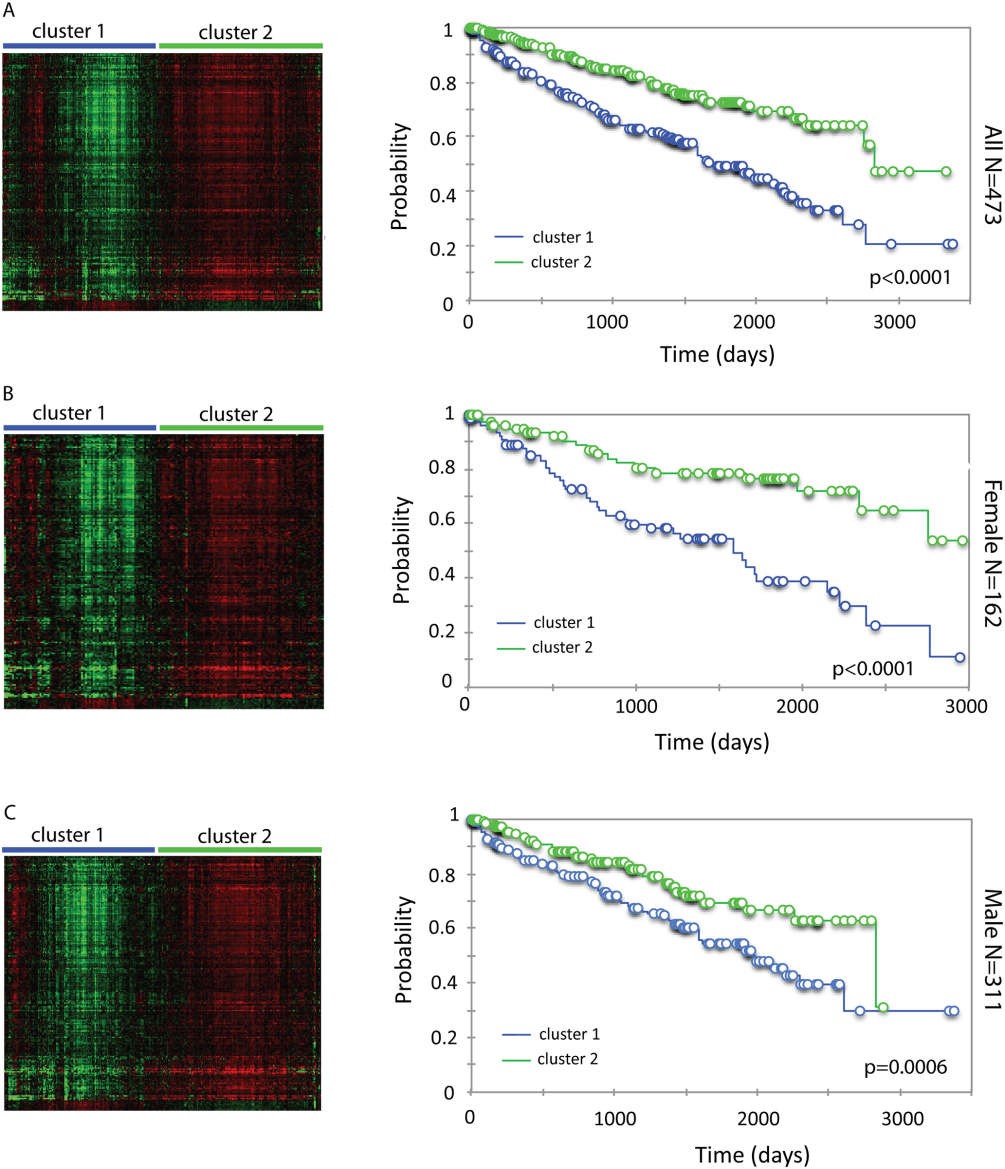
Hierarchical clustering analysis of AR-associated gene expression and its association with survival in ccRCC. (A-C, left) The heat map of AR-associated gene expression in all cases (A), females (B) and males (C). Patients were grouped into clusters based on AR-associated gene expression. (A-C, right) Kaplan-Meier analysis of AR-associated gene expression and overall survival in all cases (A), females (B) and males (C).

When 473 ccRCC patients were grouped into two clusters using hierarchical clustering analysis of expression of 199 AR-associated genes (191 positively-associated and 8 negatively- associated), patients with higher expression of genes positively-associated with AR as well as lower expression of genes negatively-associated with AR displayed significantly longer survival (Fig 5A). AR-associated gene expression similarly predicted survival in females (n = 162) (Fig 5B) and males (n = 311) (Fig 5C) analyzed separately. These results further support the conclusion that AR signaling is prognostic in ccRCC.

### Enriched pathways and upstream regulators of genes correlated with AR expression in RCC

**Fig 6:**
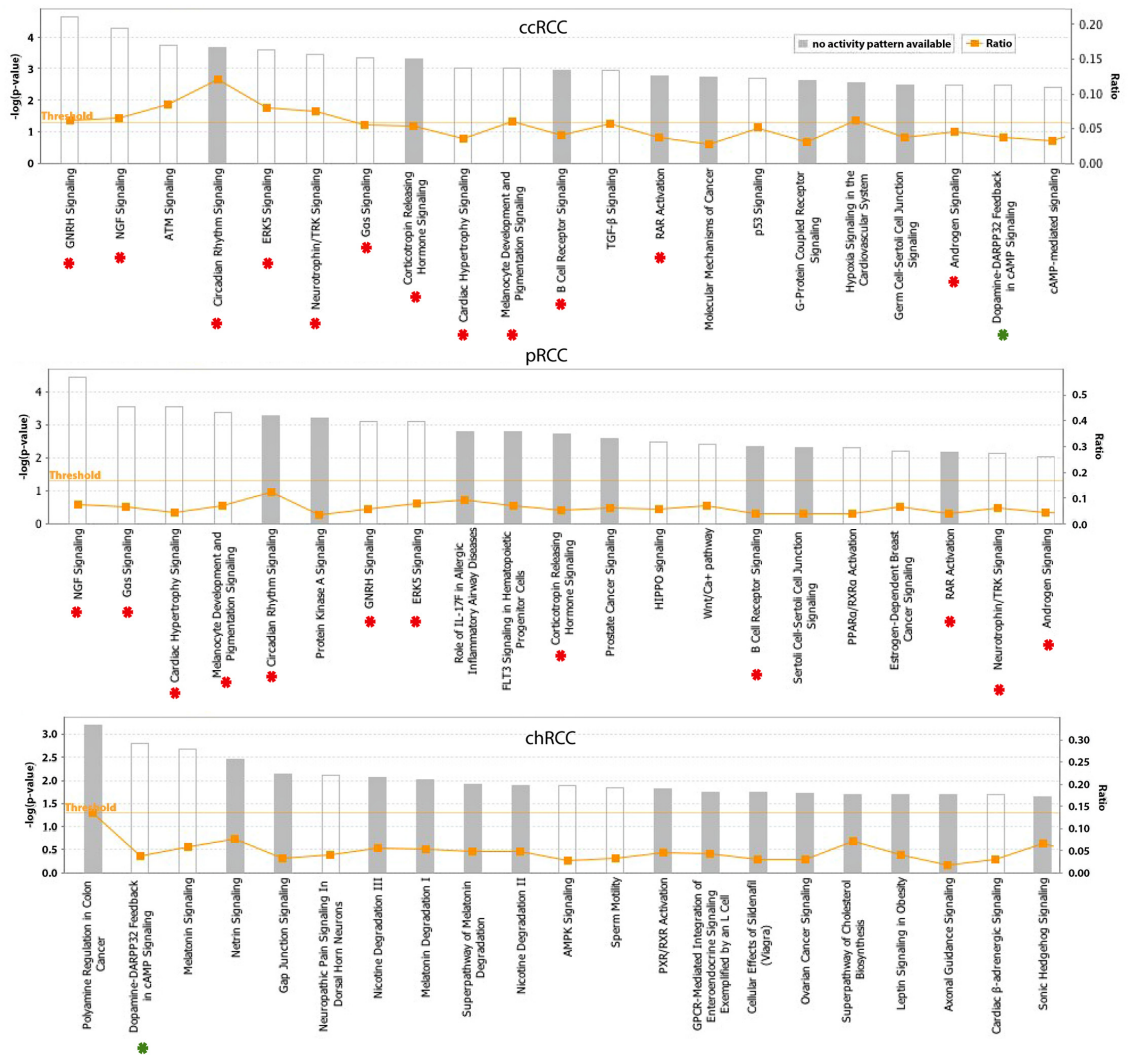
Top 21 pathways enriched in AR-associated genes identified by IPA in ccRCC, pRCC and chRCC. Red dots label pathways common in ccRCC and pRCC and green dots label pathways common in ccRCC and chRCC. No overlapping was found between pRCC and chRCC.

Pathways enriched in genes positively and negatively correlated with AR expression in each of the three RCC subtypes were identified using Ingenuity Pathway Analysis (IPA) (S2 Table). Sixty pathways were significantly associated with genes associated with AR expression in ccRCC (***p***<0.05). Forty-two and 35 pathways were enriched for pRCC and chRCC, respectively. The top 21 most significant pathways are listed in Fig 6. Twelve of these 21 pathways, including androgen signaling, overlapped between ccRCC and pRCC, and only 1 overlapped between ccRCC and chRCC. No overlap was observed between pRCC and chRCC. The number of overlapping genes in the 12 common pathways between ccRCC and pRCC is listed in Table 3. The percentage of overlap for ccRCC ranges from 63% to 100% and for pRCC from 50 to 100%, suggesting that AR regulates common pathways in ccRCC and pRCC through common target genes.

**Table 3:** The number of overlapping genes in the 12 common pathways enriched in AR-associated genes between ccRCC and pRCC.

Common pathway name	number of pathway genes in ccRCC	number of pathway genes in pRCC	number of overlapping genes	% of overlapping for ccRCC	% of overlapping for pRCC
NGF Signaling	7	8	5	71	63
G‘±s Signaling	6	7	6	100	86
Cardiac Hypertrophy Signaling	8	10	5	63	50
Melanocyte Development and Pigmentation Signaling	5	6	5	100	83
Circadian Rhythm Signaling	4	4	4	100	100
GNRH Signaling	8	7	7	88	100
ERK5 Signaling	5	5	4	80	80
Corticotropin Releasing Hormone Signaling	6	6	6	100	100
B Cell Receptor Signaling	7	7	6	86	86
RAR Activation	7	7	5	71	71
Neurotrophin/TRK Signaling	5	4	4	80	100
Androgen Signaling	5	5	5	100	100

Using IPA, the top 50 upstream regulators of genes associated with AR expression in each of the three RCC subtypes were also identified (S3 Table). Thirty-two of the top 50 upstream regulators were found in both ccRCC and pRCC and only 9 in ccRCC and chRCC. Seven were found in both pRCC and chRCC and 5 were common in all three subtypes. These results indicate that AR signaling is regulated similarly in ccRCC and pRCC, but differentially regulated in chRCC.

**Fig 7:**
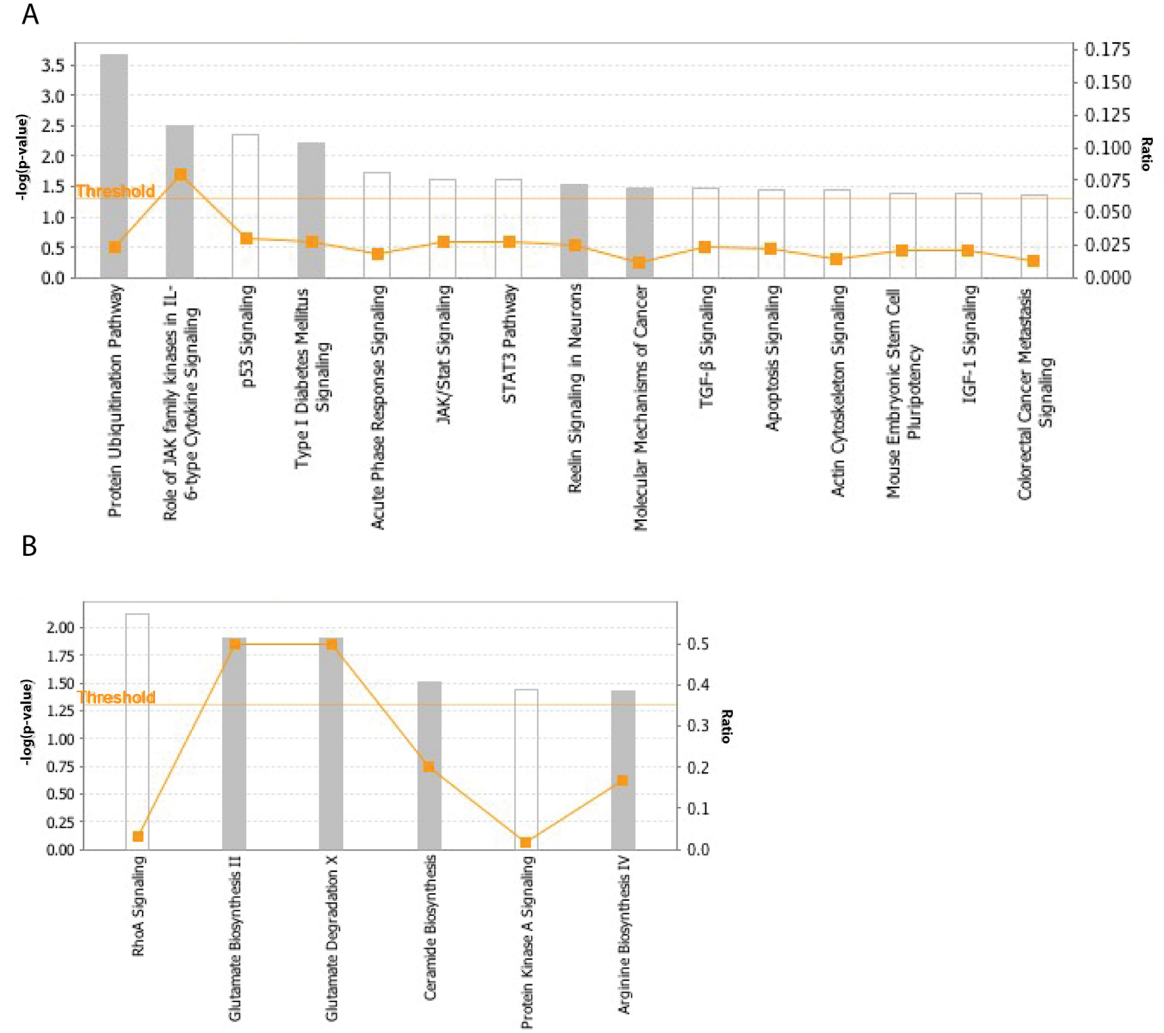
Pathways enriched in AR-associated genes identified by IPA in ccRCC but not pRCC (A) or vice versa (B).

To explore the subtype-specific role of AR in ccRCC and pRCC, we identified genes associated with AR expression only in ccRCC but not in pRCC or vice versa and pathways that were enriched in these genes. Seventy-five genes were found associated with AR in ccRCC but not in pRCC and 136 were associated with AR in pRCC but not in ccRCC (S4 Table). Fifteen pathways were significantly enriched in AR-associated genes found only in ccRCC and not pRCC (Fig 7A) and 6 were found in pRCC and not ccRCC (Fig 7B). The majority of the pathways that were enriched in ccRCC and not pRCC are known to be involved in cancer development and progression such as p53 signaling, molecular mechanisms of cancer, apoptosis signaling and colorectal cancer metastasis signaling, while a link between pathways specific to pRCC and cancer was not apparent. These results suggest that in addition to regulating common pathways in ccRCC and pRCC, AR may have a subtype-specific functionality in RCC development and progression.

### Methylation of AR in RCC

A possible mechanism for loss of AR expression in RCC is methylation. In a previous analysis of the relationship of gene expression and methylation of AR between ccRCC of stage I versus stage IV, a significant negative correlation (-0.19) was noted [18]. In our analysis of 208 cases of ccRCC with methylation and survival data available, AR expression also negatively correlated with mean methylation level of 11 sites in the AR promoter and first exon (R = -0.14). The negative association was stronger when male and female patients were analyzed separately (R = -0.32 and -0.18, respectively). Further validation is needed to confirm that DNA hypermethylation plays a causal role in the loss of AR expression.

**Fig 8:**
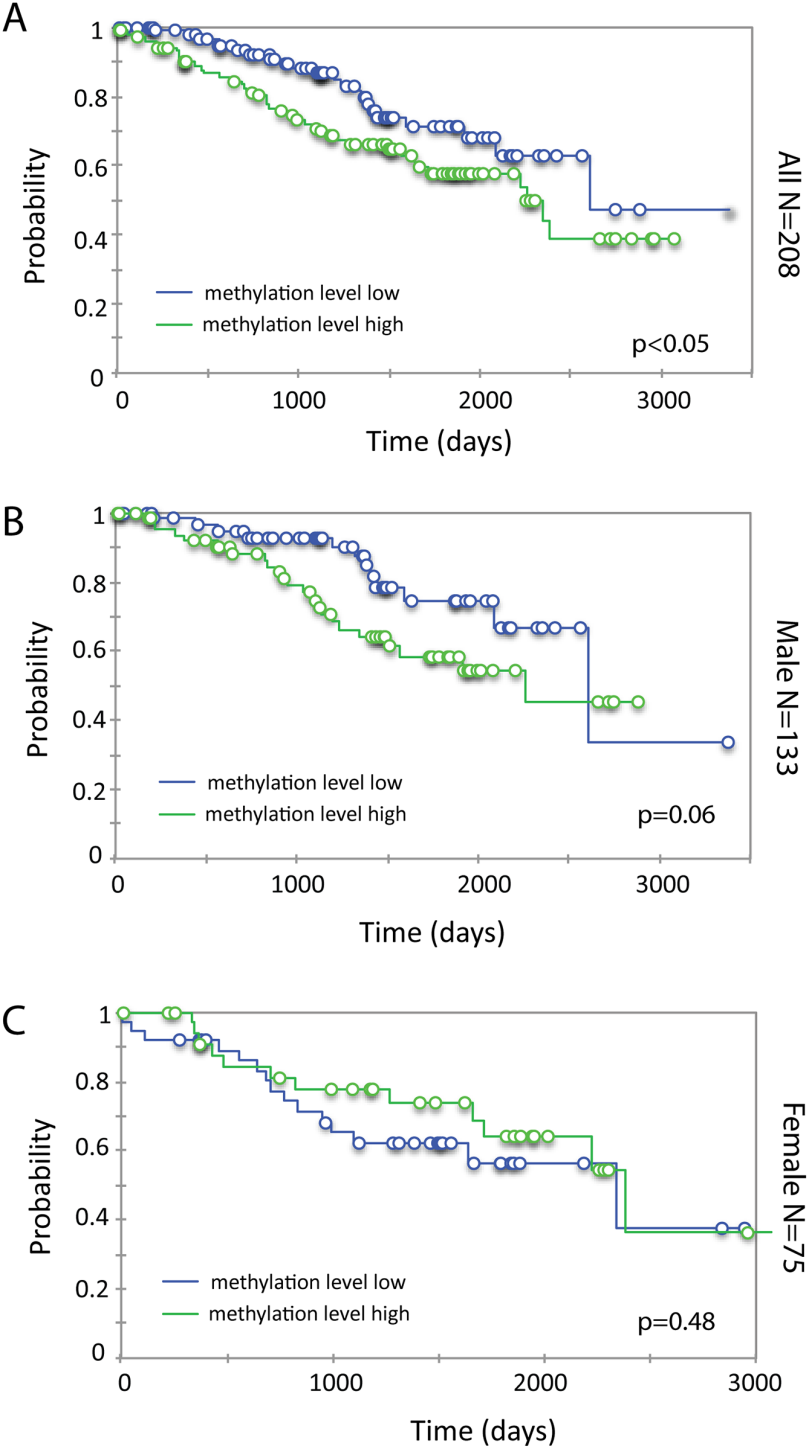
Kaplan-Meier analysis of methylation of AR promoter and first exon and overall survival in 208 ccRCC cases (A), or males (B) and females only (C). The mean methylation level of 11 CpG sites was used. Cases were assigned into methylation high or low group if their methylation level was above or below the median, respectively.

Survival analysis of 208 cases stratified by median methylation level of AR demonstrated that higher methylation was associated with poor survival (Fig 8A). When male and female patients were analyzed separately, the association of methylation level with survival was almost significant in male but not in female patients (Fig 8B and 8C), suggesting that the role of AR methylation may be different in male and female patients.

## Discussion

Our study found that AR expression in ccRCC was prognostic and that high expression predicted better overall survival in both males and females. Others have reached similar conclusions using different cohorts [9,10]. Our findings are in opposition to those from two recent studies of Korean patients with ccRCC. In those cases, AR expression associated with shorter overall survival [13,14]. However, AR positivity in one of the cohorts (63%) [14] was much higher than observed in other cohorts and the number of patients included in the second study was low (n = 57) [13]. Besides differences in patient cohorts, variations in experimental methods to determine AR expression may also contribute to the different results observed.

AR expression was variable in cases with pRCC and chRCC, but did not associate with overall survival. It is noteworthy that genes whose expression correlated with AR expression in TCGA showed a high degree of overlap between ccRCC and pRCC, but differed considerably in chRCC. Different subtypes of RCC are believed to arise in different regions of the kidney, with ccRCC and pRCC originating from the proximal tubules while chRCC arises from distal tubules [19,20]. chRCC is uncommon (~5% of RCC) and has a favorable prognosis, with low risk of tumor progression, metastases or death [19]. The unique AR-associated genes in chRCC compared to the other subtypes of RCC may indicate that the physiologic role of AR in distal tubules differs from that in proximal tubules.

In an effort to gain insight into the function of AR in RCC, we identified genes whose expression most strongly correlated with that of AR. Many of the top AR-positively correlated genes in ccRCC and pRCC have functions consistent with potential regulation by AR. For example, LMTK2 (lemur tyrosine kinase 2) had the strongest positive correlation with AR expression among the genes positively correlated with AR in both ccRCC and pRCC. This gene encodes a novel membrane-anchored kinase with a role in endosomal membrane cycling, and an essential role in spermatogenesis. Interestingly, LMTK2 negatively regulates AR expression in prostate cancer cells and decreased expression of LMTK2 has been observed in prostate cancer [21], indicating that the regulatory mechanisms between AR and LMTK2 in RCC and prostate cancer may be different. Nonetheless, the fact that 50% of AR-associated genes in ccRCC were found in ARGD and the expression of these genes predicted survival in ccRCC strongly supports the conclusion that AR signaling plays a critical role in ccRCC progression.

Loss or gain of AR function has been implicated in cancers arising from other AR-expressing tissues. Previous studies have indicated that AR may have distinct and contrasting roles in different hormone-related tumors. For example, AR contributes to the pathobiology of breast cancer [22] suggesting that inhibition of androgen signaling in breast cancer could be therapeutic [23], and in fact clinical trials are in progress to test this concept [24]. In contrast, Huang et al. demonstrated that loss of AR expression was associated with a short 5-year overall survival rate in colorectal cancer, suggesting that AR inhibited tumor progression [25]. Our analyses demonstrate that AR is protective in ccRCC and that inhibition of androgen signaling could contribute to progression of disease, suggesting that the role of AR in breast cancer might be different from that in colon cancer and RCC. Indeed, there is little overlap between genes correlated with AR expression in ccRCC in our analysis of TCGA data and those from an analysis of AR-regulated genes in breast cancer cell lines and breast tumors [26].

Whether AR has tumor suppressor or promoter activity in RCC has clinical implications. If AR is in fact a tumor suppressor, then treatment of patients bearing AR-expressing RCC with AR antagonists could be deleterious and promote, rather than prevent, progression. Conversely, treatment with AR agonists could be efficacious. In a phase II study, 30 patients with metastatic RCC received high dose testosterone proprionate plus provera, and 3 showed partial response [27]. Given that subsequent studies have shown that AR is infrequently expressed in metastatic RCC, the low response might reflect the absence of AR [10–12]. The data we presented suggest that AR is a tumor suppressor, and that devising ways to maintain or restore AR expression in RCC could have therapeutic benefit. Further, our findings suggest that loss of AR expression may sometimes occur through promoter hypermethylation and that agents that inhibit methylation might restore AR expression.

Pathways enriched in genes associated with AR expression identified by IPA analysis may provide some clues in the mechanisms of AR signaling in RCC. For example, the top common pathway affected by AR signaling in both ccRCC and pRCC is NGF signaling, which has been shown to be overexpressed in metastatic ccRCC compared to primary tumors [28]. The second-to-the-top common pathway affected by AR signaling in both ccRCC and pRCC is circadian rhythm signaling, which has been shown to regulate renal function [29]. Proteins critically involved in renal homeostatic functions have been shown to exhibit significant circadian oscillation in their expression levels or in their posttranslational modifications [30]. In addition, dysregulation of circadian rhythms is associated with the development of hypertension and accelerated progression of chronic kidney disease [31,32]. Moreover, the circadian oscillation of the mTOR pathway, important in RCC pathobiology, influenced the antitumor effect of inhibitors that target the pathway [33]. Tight interrelationships between the hypoxic response pathway and the circadian pathway have been evidenced in RCC [34]. It is intriguing to consider that AR may protect against RCC by influencing the circadian oscillations in the kidney. Further studies using realistic in vivo models such as patient-derived tissue slice grafts [35] will provide insights into the mechanisms of AR signaling in the normal human kidney and ccRCC.

## Conclusions

AR and its associated gene expression are prognostic in ccRCC with higher expression predicting longer survival. AR mediates a similar transcriptional program in ccRCC and pRCC that is different from chRCC. The tumor suppressive role of AR suggests that therapies that promote retention of AR signaling may be beneficial to ccRCC patients.

## Supporting Information

S1 Table Genes whose mRNA expression is positively or negatively associated with AR mRNA expression in each of the three RCC subtypes. (XLSX)

S2 Table Pathways enriched in genes positively and negatively correlated with AR expression in each of the three RCC subtypes identified using Ingenuity Pathway Analysis (IPA). (XLSX)

S3 Table Top 50 upstream regulators of genes associated with AR expression in each of the three RCC subtypes identified using IPA. (XLSX)

S4 Table Genes whose expression is associated with AR in ccRCC but not in pRCC and vice versa. (XLSX)
